# Predictive factors for ovarian response in a corifollitropin alfa/GnRH antagonist protocol for controlled ovarian stimulation in IVF/ICSI cycles

**DOI:** 10.1186/s12958-015-0113-1

**Published:** 2015-10-31

**Authors:** Sergio Oehninger, Scott M. Nelson, Pierre Verweij, Barbara J. Stegmann

**Affiliations:** The Jones Institute for Reproductive Medicine, Eastern Virginia Medical School, 601 Colley Avenue, Norfolk, VA 23507-2007 USA; School of Medicine, University of Glasgow, Glasgow, UK; MSD BV, Oss, The Netherlands; Merck and Co., Inc., Kenilworth, NJ USA

**Keywords:** Predictive modelling, Ovarian response, Corifollitropin alfa, GnRH antagonist

## Abstract

**Background:**

This secondary analysis aimed to identify predictors of low (<6 oocytes retrieved) and high ovarian response (>18 oocytes retrieved) in IVF patients undergoing controlled ovarian stimulation with corifollitropin alfa in a gonadotropin-releasing hormone (GnRH) antagonist protocol.

**Methods:**

Statistical model building for high and low ovarian response was based on the 150 μg corifollitropin alfa treatment group of the Pursue trial in infertile women aged 35–42 years (*n* = 694).

**Results:**

Multivariable logistic regression models were constructed in a stepwise fashion (*P* <0.05 for entry). 14.1 % of subjects were high ovarian responders and 23.2 % were low ovarian responders. The regression model for high ovarian response included four independent predictors: higher anti-Müllerian hormone (AMH) and antral follicle count (AFC) increased the risk, and higher follicle-stimulating hormone (FSH) levels and advancing age decreased the risk of high ovarian response. The regression model for low ovarian response also included four independent predictors: advancing age increased the risk, and higher AMH, higher AFC and longer menstrual cycle length decreased the risk of low ovarian response.

**Conclusions:**

AMH, AFC and age predicted both high and low ovarian responses, FSH predicted high ovarian response, and menstrual cycle length predicted low ovarian response in a corifollitropin alfa/GnRH antagonist protocol.

**Trial registration number:**

NCT01144416, Protocol P06029

## Introduction

In assisted reproductive technology, both very low and very high ovarian responses to ovarian stimulation have been associated with increased cancellation rates and compromised pregnancy and live birth rates [1, 2]. A high ovarian response also increases the risk for development of ovarian hyperstimulation syndrome (OHSS) [3]. Early identification of potential low and high responders is relevant to enable individualization of the ovarian stimulation treatment regimen [4].

The majority of studies on predictors of ovarian response have analyzed patients treated with recombinant (r) follicle-stimulating hormone (FSH) in long gonadotropin-releasing hormone (GnRH) agonist protocols. Systematic reviews have identified anti-Müllerian hormone (AMH), antral follicle count (AFC) and basal FSH as predictors of low ovarian response and AMH and AFC as predictors of high ovarian response in these protocols [5, 6], although the independence of these markers has not always been tested. AFC, basal FSH, luteinizing hormone (LH) and AMH have been identified as common prognostic factors for low or high ovarian response in rFSH GnRH antagonist protocols [7, 8].

Corifollitropin alfa is a novel recombinant gonadotropin, a single dose of which is capable of initiating and sustaining multifollicular growth during the first 7 days of ovarian stimulation as a replacement for 7 daily injections with rFSH. The treatment regimen retains the capacity for flexibility to individualize treatment after day 7 [9]. A retrospective cohort study in young women treated with corifollitropin alfa in a GnRH antagonist protocol has shown that AMH and AFC are the best predictors for low and excessive response [10].

More women are delaying pregnancy, resulting in an increased number of women over the age of 35 years seeking infertility care [11]. The Pursue trial showed that in women aged 35–42 years, a single injection of 150 μg corifollitropin alfa was noninferior to daily injections of 300 IU rFSH for the first 7 days of ovarian stimulation prior to in vitro fertilization (IVF) or intracytoplasmic sperm injection (ICSI) in terms of the vital pregnancy rate and was equally well tolerated with a low incidence of OHSS [12].

The objective of the current study was to identify predictors of low and high ovarian response in IVF/ICSI patients aged 35 to 42 years undergoing ovarian stimulation with corifollitropin alfa in a GnRH antagonist protocol, using data from the corifollitropin alfa arm of the Pursue trial.

## Materials and methods

This was a secondary analysis of data collected in the Pursue trial (*N* = 1390) (Trial registration number: NCT01144416; Protocol P06029), a double-blind, randomized controlled trial of corifollitropin alfa versus daily injections of rFSH [12]. The trial was conducted in accordance with principles of Good Clinical Practice and was approved by the appropriate institutional review boards and regulatory agencies (Chesapeake IRB, Columbia (http://www.chesapeakeirb.com/)). Written informed consent was provided by all subjects. Infertile women aged 35–42 years with a body weight of ≥50 kg and body mass index (BMI) ≥18 and ≤32 kg/m^2^ received either a single injection of 150 μg corifollitropin alfa or daily injections of 300 IU rFSH for the first 7 days of stimulation, followed by ≤300 IU/d rFSH starting on stimulation day 8, if needed. GnRH antagonist treatment, 0.25 mg/d ganirelix, was started on day 5 until final oocyte maturation with 250 μg recombinant human chorionic gonadotropin [12]. Patients were excluded if they had a history of, or current, polycystic ovary syndrome.

Validated immunoassays were performed at a central laboratory on frozen serum samples to assess FSH, LH, estradiol (E_2_) and progesterone (P) concentrations as previously described [13]. Assessment of AMH was carried out using the validated Active AMH Gen II ELISA pre-mix assay from Beckman Coulter, Inc. (Brea, California, USA).

Limits of high and low ovarian response used in the current analyses were set at >18 oocytes retrieved and <6 oocytes retrieved, respectively.

## Statistical methods

Initially, separate logistic regression models were constructed for high ovarian response (>18 oocytes retrieved or cycle canceled by the investigator because of too high response) and low ovarian response (<6 oocytes retrieved or cycle canceled due to insufficient response). Age was included as the first variable in both models. Other candidate prognostic factors were age at menarche (years), average menstrual cycle length (days), duration of infertility (years), BMI (kg/m^2^), AFC and serum levels of FSH (IU/L), LH (IU/L), E_2_ (pmol/L), P (nmol/L) and AMH (ng/mL) on day 1 of stimulation. For each candidate predictor, the association with high and low ovarian response was assessed using a χ^2^ test (the score test in a logistic regression model including only that predictor).

Multivariate logistic regression models were constructed in a stepwise fashion (*P* < 0.05 for entry and *P* > 0.05 for removal). Ten subjects with missing values (1.5 %) were excluded from model building, but were included in the estimation of the final model with missing covariate values imputed. For both outcomes the receiver operating characteristic (ROC) curve was plotted and the area under the curve (AUC, c-statistic) was calculated. This was done for the final model as well as for the intermediate models. These values were denoted ‘apparent’ AUCs. Optimism-corrected values were calculated using leave-one-out cross-validation (where the regression coefficients were re-estimated with each subject left out and then combined with the subject’s covariate values in order to mimic the prediction of the outcome for each subject). The ‘optimal’ point on the ROC curve providing the best trade-off between sensitivity and specificity and the associated ‘optimal’ probability cutoff were identified. Sensitivity, specificity, positive predictive value and negative predictive value at the optimal cutoff were calculated.

Additionally, a combined model was constructed to predict both high and low ovarian by including the predictors that appeared in both models for the separate endpoints. The impact of leaving out prognostic factors that appeared in only one of the models was investigated. In the combined model, the regression coefficient of a given factor was allowed to differ between the outcomes of high and low ovarian response (i.e., proportional odds was not assumed).

External model validation was not possible as Pursue was the only study with corifollitropin alfa for which AMH measurements were available. Instead, models were internally validated by bootstrapping [14]. A total of 500 samples from 686 were drawn with replacement from the data set analyzed and for each sample, a logistic regression model was fitted for high, and separately, for low ovarian response using the stepwise approach described above. Each model was validated using the subjects not included in the bootstrap sample (on average, 36.8 %) [15]. Validation focused on discrimination—the ability of the model to distinguish between subjects with and without the event of interest, and calibration—the correspondence between the predicted event probabilities and observed proportions. Discrimination was measured by the AUC (or c-statistic) and calibration was measured by the calibration slope. Both quantities were obtained by fitting a logistic regression model in the validation sample with a single covariate for the so-called linear predictor, a combination of covariate values (from subjects in the validation sample) and regression coefficients (from the model constructed in the bootstrap sample). The calibration slope is the regression coefficient of the linear predictor, which should be close to unity. If the calibration slope is markedly less than one, this suggests that predictions should be ‘shrunken’ toward the mean when applied to future patients. The distribution of AUCs and calibration slopes was summarized over the 500 validation samples.

Finally, we compared our models with those developed by Polyzos et al. for excessive (>20 oocytes retrieved) and poor ovarian response (<3 oocytes retrieved). It should be noted, however, that the Polyzos et al. models were based on younger women from a single European center [10].

All analyses were performed using SAS PC version 9.3 (SAS Institute Inc., Cary, NC, USA).

## Results

In this study population, 14.1 % of women were high ovarian responders (>18 oocytes retrieved) and 23.2 % were low ovarian responders (<6 oocytes retrieved). Descriptive statistics of potential predictors for ovarian response are shown in Table 1. These analyses showed that age at baseline, menstrual cycle length, AFC, FSH and AMH had a strong (*P* < 0.001) association with both high and low ovarian response.

**Table 1:** Descriptive statistics of potential predictors (covariates) for ovarian response and their univariate correlation with high and low ovarian response

Covariate	Overall	Low (<6 oocytes)	Normal (6–18 oocytes)	High (>18 oocytes)	High versus normal/low	Low versus normal/high
	(*n* = 686)	(*n* = 159)	(*n* = 430)	(*n* = 97)	(*P*-value)	(*P*-value)
Age at baseline, y, mean (SD)	38.0 (2.2)	38.6 (2.2)	37.9 (2.1)	37.3 (2.1)	<0.001	<0.001
Age at menarche, y, mean (SD)	12.9 (1.5)	12.6 (1.4)	12.9 (1.5)	13.1 (1.6)	0.097	0.026
Average menstrual cycle length, days, mean (SD)	28.2 (1.7)	27.5 (1.5)	28.3 (1.7)	28.7 (1.7)	<0.001	<0.001
Duration of infertility, y, mean (SD)	2.8 (2.8)	2.5 (2.3)	2.9 (2.8)	3.2 (3.2)	0.169	0.100
BMI at baseline, kg/m^2^, mean (SD)	25.1 (3.6)	25.1 (3.6)	25.1 (3.7)	25.0 (3.4)	0.705	0.893
AFC at day 1 of stimulation, n, mean (SD)	10.8 (4.0)	7.8 (3.1)	11.2 (3.6)	14.5 (3.4)	<0.001	<0.001
	(*n* = 679)	(*n* = 157)	(*n* = 426)	(*n* = 96)		
FSH at day 1 of stimulation, IU/L, median	6.9	8.2	6.9	6.1	<0.001	<0.001
LH at day 1 of stimulation, IU/L, median	4.6	4.2	4.7	4.7	0.555	0.089
Estradiol at day 1 of stimulation, pmol/L, median	140.6	140.2	141.5	138.4	0.163	0.981
Progesterone at day 1 of stimulation, nmol/L, median	1.9	1.9	1.9	2.0	0.984	0.849
AMH at day 1 of stimulation, ng/mL, median	1.5	0.5	1.6	3.2	<0.001	<0.001

Note: 8 subjects who did not have oocyte retrieval (for reasons other than too low or too high ovarian response according to the investigator) were excluded from the analysis

*SD* standard deviation, *BMI* body mass index, *AFC* antral follicle count, *FSH* follicle-stimulating hormone, *LH* luteinizing hormone, *AMH* anti-Müllerian hormone

### High ovarian response

**Fig. 1:**
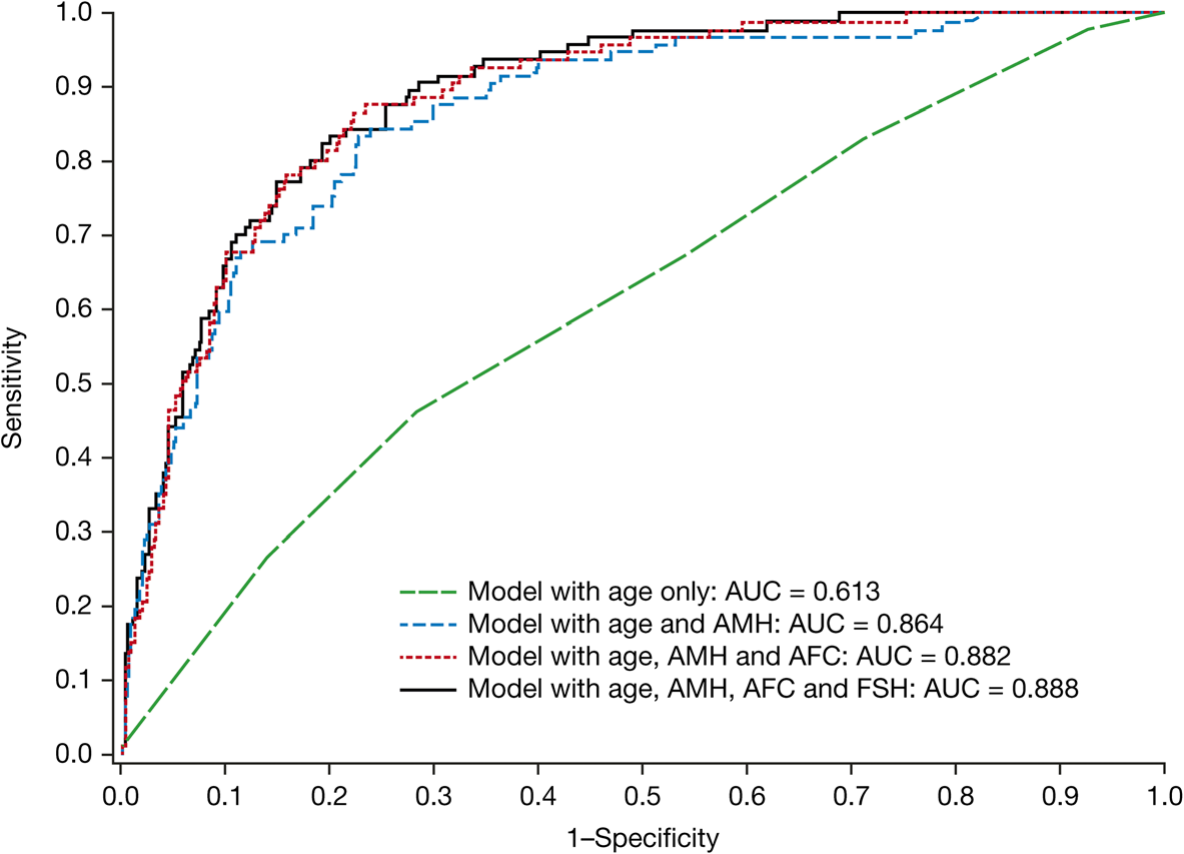
Receiver operating characteristic curves for models for high ovarian response (>18 oocytes). AUC: area under the curve; AMH: anti-Müllerian hormone; AFC: antral follicle count; FSH: follicle-stimulating hormone

The logistic regression model for high ovarian response included four independent predictors (Table 2). Higher AMH concentrations and AFCs increased the risk for high ovarian response and higher FSH levels and advancing age decreased the risk. The apparent AUC of the ROC curve for the complete model predicting high ovarian response was 0.888 (Fig. 1). Correcting for the optimism associated with measuring the performance of the model in the same data set in which the model was constructed, the AUC was 0.880 (Table 2).

**Table 2:** Logistic regression model for high ovarian response (>18 oocytes)

Covariate	OR	95 %	CI	*P*-value	AUC^a^	AUC^b^
Age (years)	0.88	0.78	1.00	0.0590	0.613	0.545
AMH (ng/mL)	1.93	1.58	2.36	<0.0001	0.864	0.858
AFC (count)	1.20	1.11	1.29	<0.0001	0.882	0.876
FSH (IU/mL)	0.78	0.65	0.93	0.0055	0.888	0.880

All odds ratios (OR) are per unit increase

*CI* confidence interval, *AUC* area under the curve, *AMH* anti-Müllerian hormone, *AFC* antral follicle count, *FSH* follicle-stimulating hormone

^a^Apparent

^b^Optimism-corrected

Table 2 and Fig. 1 also show that high ovarian response cannot be predicted by age alone (apparent AUC = 0.613). Adding AMH strongly increased the ability of the model to separate patients with high ovarian response from those without high ovarian response (AUC = 0.864). Further inclusion of AFC and FSH also increased the performance of the model, but to a lesser extent (AUC = 0.888). The sensitivity and specificity of the final model were 84 % and 80 %, respectively (Table 3). The regression equation for the final model is given in Table 3 (first row). The equation can be used to calculate the probability for high ovarian response for any patient, given her age, AMH, AFC and FSH. For a 38-year-old patient with AMH = 1.8 ng/mL, AFC = 11 and FSH = 7.5 IU/L, the linear predictor LP = −2.676 and the probability for high ovarian response is 0.064, or 6.4 %. For another 38-year-old patient with AMH = 0.8 ng/mL, AFC = 8 and FSH = 8.5 IU/L, the LP = −4.136 and the probability for high ovarian response is 0.016, or 1.6 %.

**Table 3:** Test characteristics and equations for models for high and low ovarian response

				Predictive value	
Model	Cutoff	Sensitivity	Specificity	Positive	Negative
High ovarian response^a^	0.15	0.84	0.80	0.41	0.97
Low ovarian response^b^	0.37	0.77	0.87	0.64	0.93
Combined high ovarian response^c^	0.13	0.87	0.78	0.39	0.97
Combined low ovarian response^d^	0.36	0.78	0.86	0.62	0.93

Model-based probability $P=\frac{e^{LP}}{1+{e}^{LP}}$ where LP is the linear predictor

^a^LP = 0.6953 – 0.1232 × age [years] + 0.6596 × AMH [ng/mL] + 0.1829 × AFC [count] – 0.2517 × FSH [IU/mL]

^b^LP = 5.1380 + 0.0961 × age [years] – 1.6821 × AMH [ng/mL] – 0.1690 × AFC [count] – 0.2304 × CLn [days]

^c^LP = −1.1213 – 0.1258 × age [years] + 0.7010 × AMH [ng/mL] + 0.1942 × AFC [count]

^d^LP = −0.7701 + 0.0828 × age [years] – 1.7373 × AMH [ng/mL] – 0.1635 × AFC [count]

If the model-based predicted probability is above the cutoff, a patient would be classified as a potential high (respectively, low) responder

*AMH* anti-Müllerian hormone, *AFC* antral follicle count, *CLn* cycle length, *FSH* follicle-stimulating hormone

### Low ovarian response

**Fig. 2:**
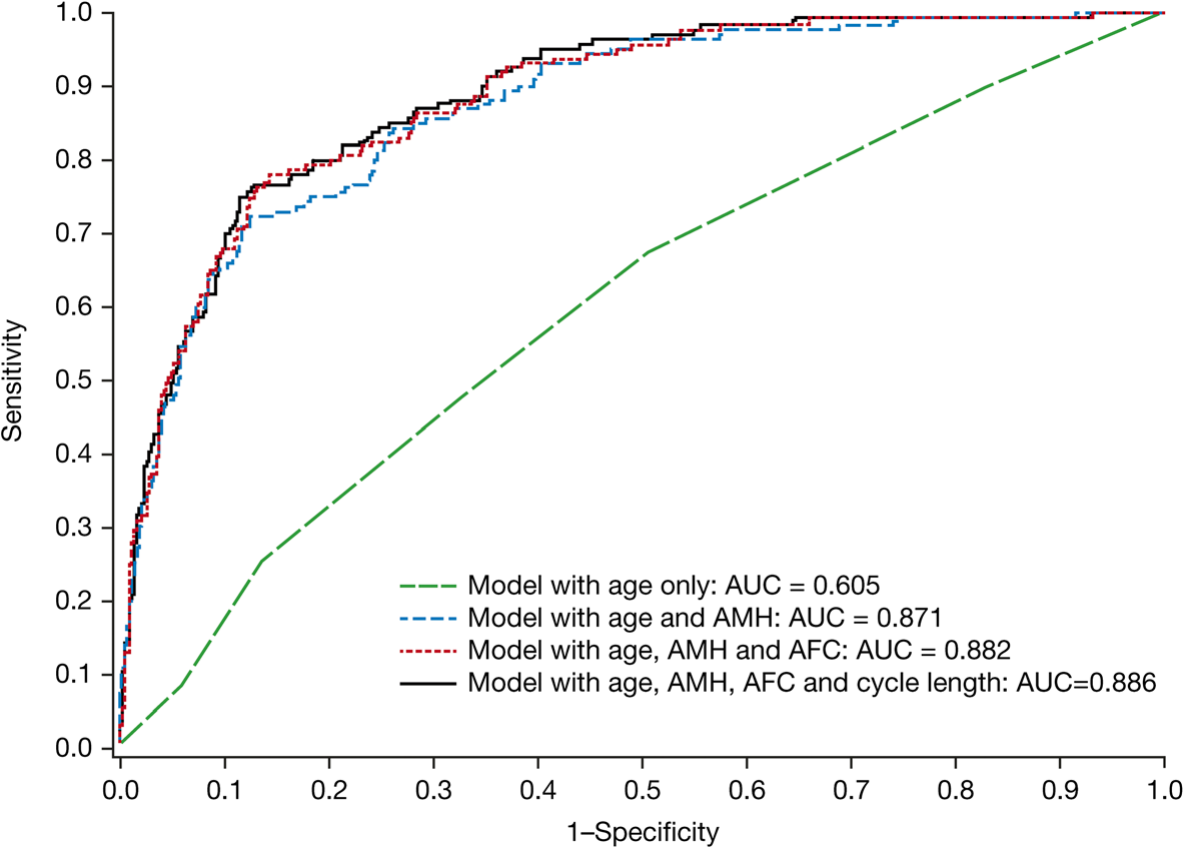
Receiver operating characteristic curves for models for low ovarian response (<6 oocytes). AUC: area under the curve; AMH: anti-Müllerian hormone; AFC: antral follicle count

The multivariable regression model for low ovarian response also included four independent predictors (Table 4). Advancing age increased the risk for low ovarian response and higher AMH, higher AFC and longer menstrual cycle length decreased the risk. The apparent AUC of the ROC curve for the complete model predicting low ovarian response was 0.886 (Fig. 2). The optimism-corrected AUC was 0.877 (Table 4).

**Table 4:** Logistic regression model for low ovarian response (<6 oocytes)

Covariate	OR	95 %	CI	*P*-value	AUC^a^	AUC^b^
Age (years)	1.10	0.99	1.22	0.0711	0.605	0.553
AMH (ng/mL)	0.19	0.12	0.28	<0.0001	0.871	0.867
AFC (count)	0.85	0.78	0.91	<0.0001	0.882	0.877
Menstrual cycle length (days)	0.79	0.69	0.92	0.0017	0.886	0.877

Odds ratios are per unit increase

*CI*, confidence interval, *AUC* area under the curve, *AMH* anti-Müllerian hormone, *AFC* antral follicle count

^a^Apparent

^b^Optimism-corrected

Table 4 and Fig. 2 again show that low ovarian response cannot be predicted by age alone (apparent AUC = 0.605). Adding AMH strongly increased the discriminative ability of the model (AUC = 0.871), whereas further inclusion of AFC and menstrual cycle length also increased the performance of the model (AUC = 0.886). The sensitivity and specificity of the final model were 77 % and 87 %, respectively (Table 3). The regression equation for the final model is given in Table 3 (second row). For a 38-year-old patient with AMH = 1.8 ng/mL, AFC = 11 and a menstrual cycle length of 28 days, the linear predictor LP = −2.548 and the probability for low ovarian response is 0.073, or 7.3 %. For the 38-year-old patient with AMH = 0.8 ng/L, AFC = 8 and FSH = 8.5 IU/L, the LP = −0.359 and the probability for low ovarian response is 0.411, or 41.1 %.

### Combined model

The regression models for high and low ovarian response had three predictors in common: age, AMH and AFC. The added value of FSH in the model for high ovarian response, although statistically significant, was not overwhelming. The same is true for menstrual cycle length in the model for low ovarian response. Without these factors, the AUC would decrease by only 0.006 and 0.004 for high and low ovarian response, respectively. Although age could also be dropped from the model without losing much predictive power, this factor was kept in the model as it is readily available. It should be noted that predicting high and low ovarian response based on the combined regression model should be based on different regression equations (Table 3, third and fourth rows). For the 38-year-old patient with AMH = 1.8 ng/mL and AFC = 11, the linear predictors for high and low ovarian response are −2.504 and −2.550, respectively and the associated probabilities are 7.6 % and 7.2 %, respectively (the estimates based on previous models were 7.3 % and 7.3 %, respectively). The remaining 85.2 % is the probability of a normal ovarian response between six and 18 oocytes.

Interpretation and application of the model would be simpler if age, AMH and AFC were classified as ‘high’ or ‘low,’ for example, by using a threshold that optimizes sensitivity and specificity for each single factor. For high ovarian response, these thresholds are age ≤37 years, AMH ≥2.24 ng/mL and AFC ≥13 (details not shown). For low ovarian response, the values are age ≥39 years, AMH ≤1.03 ng/mL and AFC ≤9. However, it is well known that dichotomization of continuous covariates leads to loss of information. Indeed, the AUC of the simpler model for high ovarian response drops to 0.867 (from 0.882) and the AUC of the simpler model for high ovarian response drops to 0.841 (also from 0.882). For this reason, the simpler models were not pursued further.

### Model validation

The bootstrap validation of the model for high ovarian response (including variable selection) resulted in a median AUC of 0.895 in the validation samples (2.5 and 97.5 percentage points: 0.862 and 0.923). The median calibration slope was 0.990 (0.964–1.004). The validation of the model for low ovarian response based on the same 500 bootstrap samples resulted in a median AUC of 0.890 (0.857–0.917) and a median calibration slope of 0.937 (0.557–1.461). These results suggested good discrimination and calibration for high and low ovarian response.

### Comparison with the model of Polyzos et al.

Our findings agree with those of Polyzos et al. [10] in that AMH and AFC are important predictors of ovarian response. Polyzos et al. developed models for excessive ovarian response (>20 oocytes retrieved) and poor ovarian response (<3 oocytes retrieved) based on AMH and AFC. The linear predictor for excessive ovarian response based on our data would be LP = – 7.287 + 0.664 × AMH + 0.260 × AFC (details not shown), rather similar to the regression equation of Polyzos et al. (z = – 6.782 + 0.557 × AMH + 0.172 × AFC).

Our linear predictor for poor ovarian response, LP = 0.964 – 2.710 × AMH – 0.167 × AFC, however, is different from their regression equation (z = 2.161 – 0.991 × AMH – 0.171 × AFC). The main reason is that the percentage of poor responders in our data set is markedly lower than reported by Polyzos et al. (6.7 % versus 34.3 %). This difference could not be accounted for by possibly different values of AMH and AFC in our population. The percentages of excessive response were similar (9.5 % versus 8.6 %). Using the linear predictors of Polyzos et al. in our data resulted in calibration slopes of 1.31 and 1.72 for excessive and poor response, respectively. These were statistically significantly different from one (*P* = 0.032 and *P* = 0.0032, respectively), suggesting that these predictors should not be used for older women. Predictive models inevitably reflect the data set from which they are derived, which is why we made the validation effort.

## Discussion

In the population of women in the Pursue trial aged 35–42 years old undergoing ovarian stimulation with corifollitropin alfa in a GnRH antagonist protocol, the predictive factors for high ovarian stimulation were age, AFC, basal FSH and AMH, factors previously identified from rFSH GnRH agonist and antagonist protocols [5–8]. In the older patient population in the Pursue trial [12], increased menstrual cycle length was identified as a factor that decreased the risk of low ovarian response. Women with a history of polycystic ovary syndrome were excluded. Compared with younger women (18–36 years old) undergoing ovarian stimulation with rFSH in a GnRH antagonist protocol, in the current analyses, increased LH and BMI [16] were not identified as predictors of high ovarian response [8].

In this study, AMH concentrations were measured on frozen serum samples using the Active AMH Gen II ELISA pre-mix assay. For stored samples, there is no difference between the pre-mix and post-mix protocols in AMH concentrations, as complement degradation has already occurred resulting in minimal interference in the assay [17].

Limits of high (>18 oocytes retrieved) and low (<6 oocytes retrieved) ovarian response used in the current analyses were selected as subjects with more than 18 oocytes recovered have an increased risk of OHSS [3] and those with fewer than six oocytes recovered have a compromised chance of pregnancy [1, 2]. These limits are consistent with previous publications on excessive and low ovarian response [7, 8].

In a retrospective analysis of a prospective randomized trial in which patients aged 18–36 years were treated with corifollitropin alfa or rFSH in a GnRH antagonist protocol, the ongoing pregnancy success rates in high responders (186 subjects with >18 oocytes) were at least as high as in patients with fewer than 18 oocytes [18]. This difference from the Sunkara analysis [1] may be related to the fact that these were women with a normal menstrual cycle and body weight range, and those with extremes in AFC and with polycystic ovary syndrome were excluded. Also, the Sunkara analysis was mainly an analysis of GnRH agonist cycles [1].

The current analyses of older women undergoing ovarian stimulation with corifollitropin alfa in a GnRH antagonist protocol uphold that age of the subject, AFC and AMH are strong predictors of ovarian response, as indicated in younger women undergoing ovarian stimulation with rFSH in a GnRH agonist or antagonist protocol.

We conclude that in women aged 35 to 42 years undergoing ovarian stimulation with corifollitropin alfa in a GnRH antagonist protocol, AMH, AFC and age at the start of stimulation were prognostic for both high and low ovarian response, in addition to FSH for high ovarian response and menstrual cycle length for low ovarian response.

## Abbreviations

AFC: Antral follicle count
AMH: Anti-Müllerian hormone
AUC: Area under the curve
BMI: Body mass index
E_2_: Estradiol
FSH: Follicle-stimulating hormone
GnRH: Gonadotropin-releasing hormone
ICSI: Intracytoplasmic sperm injection
IVF: In vitro fertilization
LH: Luteinizing hormone
OHSS: Ovarian hyperstimulation syndrome
P: Progesterone
r: Recombinant
ROC: Receiver operating characteristic
